# The role of inter-regional mobility in forecasting SARS-CoV-2 transmission

**DOI:** 10.1098/rsif.2022.0486

**Published:** 2022-08-31

**Authors:** Martijn H. H. Schoot Uiterkamp, Martijn Gösgens, Hans Heesterbeek, Remco van der Hofstad, Nelly Litvak

**Affiliations:** ^1^ Department of Mathematics and Computer Science, Eindhoven University of Technology, Eindhoven, The Netherlands; ^2^ Department of Population Health Sciences, Utrecht University, Utrecht, The Netherlands; ^3^ Faculty of Electrical Engineering, Mathematics and Computer Science, University of Twente, Enschede, The Netherlands

**Keywords:** inter-regional mobility, forecasting, epidemiology, compartmental models, SARS-CoV-2

## Abstract

In this paper, we present a method to forecast the spread of SARS-CoV-2 across regions with a focus on the role of mobility. Mobility has previously been shown to play a significant role in the spread of the virus, particularly between regions. Here, we investigate under which epidemiological circumstances incorporating mobility into transmission models yields improvements in the accuracy of forecasting, where we take the situation in The Netherlands during and after the first wave of transmission in 2020 as a case study. We assess the quality of forecasting on the detailed level of municipalities, instead of on a nationwide level. To model transmissions, we use a simple mobility-enhanced SEIR compartmental model with subpopulations corresponding to the Dutch municipalities. We use commuter information to quantify mobility, and develop a method based on maximum likelihood estimation to determine the other relevant parameters. We show that taking inter-regional mobility into account generally leads to an improvement in forecast quality. However, at times when policies are in place that aim to reduce contacts or travel, this improvement is very small. By contrast, the improvement becomes larger when municipalities have a relatively large amount of incoming mobility compared with the number of inhabitants.

## 1. Introduction

In the effort to contain a pandemic, local contact-restricting measures tailored to particular regions within a country can be beneficial when those regions exhibit large differences in infection prevalence. For instance, local measures can be implemented specifically for regions with a high level of infections, whereas such measures are not necessary for other regions with lower levels of infections. To take such decisions on a regional level, the local policymakers need information on the effect that measures have on their specific region. Therefore, for such a region-focused approach to be successful, it is important to know the effect of measures on the progression of infections on this local level, rather than only on the national level.

Since the start of the COVID-19 pandemic, a substantial number of studies have focused on forecasting the course of the pandemic, for example to be able to judge the effect of policy changes (see, for example, the overview in [1]). Already at the very start of the pandemic, the role of human mobility has been studied extensively, establishing a correlation between reductions in mobility (e.g. as a consequence of national restrictive measures) and the spread of the virus (e.g. [2–4]). A general conclusion from these studies is that human mobility between regions contributes significantly to the initial transmission and spread of the virus. Similarly, a reduction in mobility as a consequence of restrictive measures is shown to correlate with a reduction of new infections. This suggests that information on the influence of inter-regional mobility on the virus spread is useful for regional policymakers.

When focusing on the influence of mobility on the spread of the virus, it is important to identify which type of mobility is most relevant and which level of granularity and detail in mobility information is required. On the one hand, the country-level may be sufficient for inference and forecasting the general development of the pandemic, but is not suitable to gain insight into the emergence of local outbreaks or super-spreading events, which have been shown to be important [5,6]. On the other hand, small-scale mobility, for example, on the level of individuals, can provide enormous insight into demographic aspects of the transmission process [7], but require many assumptions on the behaviour of individuals and such assumptions are generally hard to validate [8,9]. Another reason why determining the right level of detail is important, is that exact mobility information is not available for reasons of privacy. Thus, proxies must be used instead, i.e. different data that approximate the desired data or can be used to obtain such an approximation. The availability and accuracy of such proxies depends on the level of granularity and detail, where smaller-scale and more detailed proxies are generally less readily available and more sensitive to noise.

A perhaps even more fundamental question is: under which epidemiological conditions and restrictive measures does incorporating mobility in transmission models actually yield significant improvements in estimating the state of the epidemic and forecasting its development? For instance, several studies [10–12] found a strong correlation between changes in mobility and infection prevalence at the start of the pandemic, when a severe lockdown was in place. However, after the first wave, this correlation was much weaker, presumably because different restrictive measures were in place that did not directly limit the number of contact moments (e.g. an advise to work from home instead of the closing of workplaces). This suggests that changes in mobility are not always predictive of changes in infection incidence, which justifies the question in which epidemiological situations the inclusion of mobility yields more accurate estimations and forecasts.

The goal of this paper is to develop methodological tools to ultimately support regional policymakers in taking appropriate contact-restrictive measures to contain the spread of SARS-CoV-2, where we take the epidemic in The Netherlands as a use-case. For this, we study the influence of inter-regional mobility on the spread of infections and aim to answer the following two questions:

1. How effective are particular types of measures in reducing the number of local and inter-regional contacts? To answer this question, we estimate these numbers of contacts for our case study of the situation in The Netherlands using a mobility-enhanced SEIR-compartmental model [3]. We find that measures that restricted visiting public places in August 2020 and a set of measures representing a partial lockdown in October and November 2020 (including the closing of bars and restaurants and a strong advice to work from home) were followed by a reduction in both the number of local and inter-regional contact moments. However, further restricting contacts by additional measures, such as minimizing the number of allowed guests at home and the closing of public places in November 2020, was followed by an increase in local contacts.
2. When does taking inter-regional mobility into account improve the quality of short-term forecasts of the spread of infection? We answer this question by comparing the forecast quality of the aforementioned model to that of the same model without mobility. We find that taking mobility into account generally improves the forecast quality of the model. However, under measures that aim to reduce the amount of commuter travel and work contacts, this improvement turns out to be insignificant. This suggests that, under such measures, information on this type of travel is not necessary to explain the development of new infections. Moreover, we find evidence that suggests a larger improvement for municipalities where the share of incoming mobility is large compared with the number of inhabitants. The overall forecast accuracy itself is worse for regions that are known for having more contacts than average, that have a large share of foreign travel (which we do not explicitly take into account), or that have had disproportionally many positive cases.

We build in this work on the mobility-enhanced SEIR-compartmental model of [3], where a given area is divided into several smaller regions (e.g. a country that is divided into municipalities). Transmissions within a given region are modelled using a standard SEIR-compartmental model [13] and transmissions caused by contacts with inhabitants from other regions are modelled by enhancing the standard formulation of the SEIR-model with an additional term involving the mobility between these regions and their susceptible and infectious populations. Potential differences between the average number of intra- or inter-regional contacts are modelled via a distinct transmission rate for both contact types.

Several parameters of this model, such as the average latent and infectious periods, can be taken from the literature since they concern the intrinsic properties of the virus that are not location-dependent. However, the transmission rates are dependent on the number of contact opportunities and are time-dependent. In earlier work [3], these parameter values were chosen based on the literature available at that time with the goal to serve as illustrative examples when assessing the trade-off between mobility restrictions and virus transmission. In several studies in the literature, a maximum-likelihood estimation (MLE) approach is followed to estimate the single transmission rate for compartmental models that do not include mobility (see [14–16] and references therein). In the current work, we develop an MLE procedure to determine both transmission rates in the mobility-enhanced model of [3], simultaneously based on the infection prevalence of SARS-CoV-2 in the Dutch population. To do this, we turn the corresponding difference equations of the model into a partially *stochastic* model, where one of the difference equations is interpreted as the parameter of a suitable negative binomial random variable.

There are many studies that focus on the additional value of taking mobility into account when investigating the spatial prevalence and transmission dynamics of infectious diseases [17,18], thereby also stressing the importance of choosing the right proxy for mobility data, given the desired accuracy [19]. For the specific case of the COVID-19 pandemic, various models and mobility proxies have been considered. These models range from data-driven models with minimal assumptions on transmission dynamics [20,21] to process-focused approaches that incorporate mobility in existing transmission models, such as used in this paper (see also [22]). Data used to estimate mobility patterns primarily consist of origin-destination data between regions obtained from social media platforms [21,23] or mobile phone operators [24]. Compared to these works, the novelty of our work is that we directly compare transmission models that do and do not include mobility and investigate when and where including mobility improves the model and forecast accuracy.

Our evaluations indicate that the mobility-enhanced model and estimation procedure yield accurate forecasts of the distribution of infections throughout the country, i.e. the percentage of all reported infections that occurs in each region. In particular, we obtain accurate forecasts of the *order* of regions in terms of their share in the total number of reported infections. This suggests that the model and the mobility data are sophisticated enough to describe the inter-regional patterns in virus transmission. This is important information for regional policymakers since they are interested in the potential sources of local COVID-19 outbreaks. However, we also find that the model is able to accurately forecast the *volume* of infections only during longer periods without changes in mobility-restricting measures. The predictive quality of the model reduces significantly within the first week after such changes but, in the subsequent week, returns to the original degree of accuracy. The main cause of this reduction in accuracy is the absence of information on the most recently infected individuals.

In this paper, we focus primarily on spread in The Netherlands. However, we believe that our general conclusions with regard to, for example, the added value of accounting for mobility also apply to similar countries, i.e. densely populated countries with substantial inter-regional mobility. Furthermore, although we focus here on the SARS-CoV-2 pandemic, our approach is also applicable to other infectious diseases with similar epidemiological characteristics.

The organization of the paper is as follows. In §2, we introduce and explain the compartmental model and in §3, we explain how we initialize the parameters of this model and which data sources we use for this. In §4, we describe how we estimate the transmission rates using MLE, how we validate the model, and how we use it to investigate the influence of mobility. In §5, we present the results and, finally, in §6 we discuss the limitations of our work and state our conclusion.

## 2. The compartmental model

We mathematically describe the spread of infection using the compartmental model introduced in [3]. In this section, we provide a brief description of the model, and more details are given in electronic supplementary material, appendix A.

We divide the population of size *N* into a set *D* of separate regions and denote the population of each area *i* ∈ *D* by *N*_*i*_. We focus primarily on the division in municipalities in this paper, but in the general model divisions based on other criteria are possible. Throughout, we assume that the population remains constant over time. At each time *t*, the population of each region *i* is partitioned into six compartments that indicate their epidemiological state, denoted by $\left(S_{i}(t),E_{i}(t),I_{i}^{T}(t),I_{i}^{U}(t),R_{i}^{T}(t),R_{i}^{U}(t)\right)$. These compartments contain all susceptible (not infected), exposed (infected but not yet infectious), positively tested infectious, untested infectious, recovered positively tested and recovered untested individuals, respectively. In our model, we distinguish between positively tested and untested individuals for two reasons. First, we assume that positively tested individuals do not travel to, or receive visitors from, other regions. Second, the compartment of positively tested infectious persons is the only compartment whose size we can accurately measure or estimate.

We model mobility between regions as follows. For each two regions *i*, *j* ∈ *D*, we let the parameter *M*_*ij*_ denote the number of individuals traveling from *i* to *j* per time unit. We assume that travelling individuals visit only one region and return directly afterwards to their home region.

In compartmental epidemiological models, the transmission rate, usually denoted by *β*, determines the rate at which susceptible individuals are infected by infectious ones. With the inclusion of mobility between regions, two separate transmission rates *β*_loc_ and *β*_mob_ are introduced that correspond to the transmission rates for infections caused by intra-regional contacts and inter-regional contacts, respectively.

The compartmental model is described by the following differential equations, for each *i* ∈ *D*:

2.1 $$\begin{array}{rl} \frac{dS_{i}(t)}{dt} & =-\beta_{\mathrm{loc}}\frac{S_{i}(t)}{N_{i}}(I_{i}^{T}(t)+I_{i}^{U}(t))\\ & \;-\beta_{\mathrm{mob}}\sum_{\;j\in D}\left(S_{i}(t)\frac{M_{\;ji}}{N_{i}}\frac{I_{j}^{U}(t)}{N_{j}}+I_{j}^{U}(t)\frac{M_{ij}}{N_{j}}\frac{S_{i}(t)}{N_{i}}\right), \end{array}$$

2.2 $$\begin{array}{rl} \frac{dE_{i}(t)}{dt} & =\beta_{\mathrm{loc}}\frac{S_{i}(t)}{N_{i}}(I_{i}^{T}(t)+I_{i}^{U}(t))\\ & \;+\beta_{\mathrm{mob}}\sum_{\;j\in D}\left(S_{i}(t)\frac{M_{\;ji}}{N_{i}}\frac{I_{j}^{U}(t)}{N_{j}}+I_{j}^{U}(t)\frac{M_{ij}}{N_{j}}\frac{S_{i}(t)}{N_{i}}\right)-\frac{E_{i}(t)}{\nu },\\ \frac{dI_{i}^{T}(t)}{dt} & =a\frac{E_{i}(t)}{\nu }-\frac{I_{i}^{T}(t)}{\omega },\\ \frac{dI_{i}^{U}(t)}{dt} & =(1-a)\frac{E_{i}(t)}{v}-\frac{I_{i}^{U}(t)}{\omega },\\ \frac{dR_{i}^{T}(t)}{dt} & =\frac{I_{i}^{T}(t)}{\omega },\\ \frac{dR_{i}^{U}(t)}{dt} & =\frac{I_{i}^{U}(t)}{\omega }. \end{array}$$

Here, *a* is the fraction of infectious individuals that is positively tested, *ν* is the average latent period, i.e. the time between getting infected and becoming infectious, and *ω* is the average infectious period, during which an infected individual can infect others.

Given the probability ɛ that a transmission occurs during an appropriate contact between a susceptible and an infectious person, the overall average contact rate *c*, and the fraction *p* of those contacts that occur locally, i.e. that are not the result of inter-regional travel, we can decompose *β*_loc_ and *β*_mob_ as

2.3 $$\beta_{\mathrm{loc}}=\varepsilon pc$$

and

2.4 $$\beta_{\mathrm{mob}}=\varepsilon (1-p)\frac{cN}{2\sum_{(i,j)\in D\times D}M_{ij}}.$$

In the latter expression, the term

$$(1-p)\frac{cN}{2\sum_{i,j\in D\times D}M_{ij}}$$

represents the total number of non-local contacts (1 − *p*)(*cN*/2) divided by the total number of travels $\sum_{(i,j)\in D^{2}}M_{ij}$, i.e. the number of contacts per traveling person per unit time.

A concise explanation of the model parameters is given in table 1. Further details of the model and parameters are the same as in our earlier work [3] and are provided in electronic supplementary material, appendix A. One of the novel contributions of this work is the initialization and estimation procedure of the model parameters, based on the available data, which we address in §§3 and 4.1.

**Table 1.** Model parameters.

parameter	meaning
*N*	total population size
*D*	set of region indices
*N*_*i*_	population size of region *i*
*M*_*ij*_	number of individuals that travel from region *i* to region *j*
*a*	fraction of infectious people that have been tested
*ω*	infectious period
*v*	latent period
*β*_loc_	transmission rate via local contacts
*β*_mob_	transmission rate via non-local contacts
ɛ	transmission probability per contact between susceptible and infectious individual
*c*	average number of contacts
*p*	fraction of local contacts

## 3. Parameter initialization and data sources

The demographic parameters *D* and *N*_*i*_ are initialized based on the division of the country into regions. For instance, if we investigate on the municipality-level, then *D* contains an index for each municipality. Furthermore, the values for the average latent and infectious periods *ν* and *ω* are chosen based on the literature as 3 days [25,26] and 9 days [27–29], respectively.

To initialize the sizes of the six compartments on a given day *t* for a given municipality *i*, we use information on daily reported cases as reported by, for example official national health sources such as the Dutch National Institute for Public Health and the Environment (RIVM) [30]. More precisely, we use as input the number of positive tests reported at time *t* in region *i*, denoted by Δ*I*_*i*_(*t*), for each time *t* and region *i*. We assume that recipients of a positive test result become infectious at the report date of the test since we do not have (access to) any other information on when a positively tested individual has been infected, underwent a test, or recovered. We refer to electronic supplementary material, appendix B for the mathematical description of the initialization procedure.

For the initialization of the mobility parameters *M*_*ij*_, we combine two different data sources: one on mobility patterns from before the pandemic and the other on the relative change in mobility since the start of the pandemic. Clearly, both the volume and structure of mobility patterns has changed significantly since the start of the pandemic [31]. In particular, both the total number of travel movements and the share of long-distance trips among the total number of movements have significantly decreased [32].

The first source is data on places of residence and of work as collected by Statistics Netherlands (CBS) [33]. The second source is data that represent the change in mobility for different types of mobility compared to a pre-pandemic baseline as collected by Google [34]. We refer to electronic supplementary material, appendix C for more details on these data and how we combine them to obtain a proxy for the mobility matrix *M*.

Via studies on the seroprevalence of SARS-CoV-2 in the population, the number of hospitalizations, and the daily fraction of positive tests, estimates can be made of the total number of infectious people $I^{\mathrm{tot}}(t)\;:=\sum_{\;j\in D}(I_{j}^{T}(t)+I_{j}^{U}(t))$ at time *t*. Based on such an estimate ${\hat{I}}^{\mathrm{tot}}$ and the total number of positively tested individuals as initialized in §3, we can obtain an estimate on the fraction of positively tested infectious individuals *a* on a given day *t* as

$$a=\frac{\sum_{\;j\in D}I_{j}^{T}(t)}{\sum_{\;j\in D}(I_{j}^{T}(t)+I_{j}^{U}(t))}=\frac{\sum_{i\in D}\int_{0}^{\infty }\;e^{-(s/\omega )}\Delta I_{i}(t-s)\;ds}{{\hat{I}}^{\mathrm{tot}}(t)}.$$

For *I*^tot^(*t*), we use estimates by the RIVM based on the outcomes of the ‘Pienter Corona-studie’ [35], which investigated the seroprevalence of SARS-CoV-2 in The Netherlands (see also [36]).

Given the transmission rates *β*_loc_ and *β*_mob_, we estimate the fraction of local contacts *p* as follows (we describe how we estimate the transmission rates in §4.1). Note that, by the definition of *β*_loc_ and *β*_mob_ in equations (2.2) and (2.3),

$$\frac{\beta_{\mathrm{loc}}}{p}=\varepsilon c=\frac{2\sum_{(i,j)\in D\times D}M_{ij}}{(1-p)N}\beta_{\mathrm{mob}},$$

provided that *p* ≠ 0 and *p* ≠ 1. From this, it follows that

$$\frac{1}{p}=\frac{2\sum_{(i,j)\in D\times D}M_{ij}}{N}\frac{\beta_{\mathrm{mob}}}{\beta_{\mathrm{loc}}}+1$$

and thus

$$p=\frac{N\beta_{\mathrm{loc}}}{2\beta_{\mathrm{mob}}\sum_{(i,j)\in D\times D}M_{ij}+N\beta_{\mathrm{loc}}}.$$

Observe that we can now compute the term ɛ*c* as ɛ*c* = *β*_loc_/*p*. We do not initialize the parameters ɛ and *c* separately since they do not occur separately in our model but only together in the form ɛ*c*.

## 4. Approach

In this section, we describe how we use the compartmental model and the data initialization procedure to accomplish the following:

1. estimate the transmission rates *β*_loc_ and *β*_mob_;
2. validate the performance of the model at the nationally aggregated level;
3. assess the forecasting quality of the model on the municipality level;
4. investigate the influence of mobility on the forecast quality.

We discretize the model so that it matches the discrete-time format of the available input data, meaning among others that we look ahead 7 days and look back 14 days when initializing compartments (see electronic supplementary material, appendix D).

We focus on the developments within the second half of 2020, i.e. within the time period 1 July–31 December 2020. The reason for this is that, on the one hand, testing for the virus became available for the entire population slightly before 1 July 2020. Thus, from this time on, the daily reported cases provide a more accurate view of the number of known infections. On the other hand, mass vaccination of the population started in January 2021. Thus, the current version of our compartmental model is especially suitable for the time period 1 July 2020–31 December 2020.

### 4.1. Estimating the transmission rates *β*_loc_ and *β*_mob_

We develop an MLE procedure to estimate for a given day *t* the corresponding transmission rates. This means that we assume that for a given day *t* and municipality *i* the number of new infections on the next day, i.e. −Δ*S*_*i*_(*t*) := *S*_*i*_(*t*) − *S*_*i*_(*t* + 1), is a random variable with given distribution. The goal is to find the parameter values of this distribution that make this assumption most likely.

In particular, in line with other work on MLE for estimating transmission rates in compartmental models (e.g. [37]), we consider a commonly used model for the distributions of −Δ*S*_*i*_(*t*), namely the negative binomial distribution. Its mean is given by the negative of the right-hand side of equation (2.1), i.e. by

4.1 $$\begin{array}{rl} \lambda_{i}(t)\;: & =\beta_{\mathrm{loc}}\frac{S_{i}(t)}{N_{i}}(I_{i}^{T}(t)+I_{i}^{U}(t))\\ & \;+\beta_{\mathrm{mob}}\sum_{\;j\in D}\frac{S_{i}(t)}{N_{i}}\frac{I_{j}^{U}(t)}{N_{j}}(M_{\;ji}+M_{ij}), \end{array}$$

and its variance is given by *λ*_*i*_(*t*)(1 + (*λ*_*i*_(*t*)/*r*(*t*)))^*r*(*t*)^, where *r*(*t*) is a to-be-estimated dispersion parameter. Note that the negative binomial distribution models overdispersion since it can be interpreted as a mixed Poisson random variable whose parameter has a gamma distribution and therefore is especially suitable for modelling SARS-CoV-2 transmission [38].

Note that only the flow from *S*_*i*_(*t*) to *E*_*i*_(*t*) is taken as a stochastic variable and not the flows between other compartments as is common in some other works (e.g. [39]). The main reason for this is that these flows depend on parameters that are necessary for the compartment initialization and must therefore be estimated beforehand in a different way. Thereby, the total population size remains constant for each municipality.

The maximum-likelihood approach relies on estimating parameters by optimizing an appropriate (log-)likelihood function, which is given by

4.2 $$\begin{array}{rl} & L_{\mathrm{NB}}\;(\beta_{\mathrm{loc}},\beta_{\mathrm{mob}},r|x_{\mathrm{loc}}(t),x_{\mathrm{mob}}(t),\Delta S(t))\\ & \;=\sum_{i\in D}\log(\frac{(\beta_{\mathrm{loc}}x_{i,\mathrm{loc}}(t)+\beta_{\mathrm{mob}}x_{i,\mathrm{mob}}{\left(t)\right)}^{-\Delta S_{i}(t)}}{(-\Delta S_{i}(t))!}\\ & \;\;\times \frac{\Gamma (r-\Delta S_{i}(t))}{\Gamma (r){\left(r+\beta_{\mathrm{loc}}x_{i,\mathrm{loc}}(t)+\beta_{\mathrm{mob}}x_{i,\mathrm{mob}}(t)\right)}^{-\Delta S_{i}(t)}}\\ & \;\;\times \frac{1}{{\left(1+(\left(\beta_{\mathrm{loc}}x_{i,\mathrm{loc}}(t)+\beta_{\mathrm{mob}}x_{i,\mathrm{mob}}(t)\right)/r)\right)}^{r}})\\ & \;=\sum_{i\in D}(-\Delta S_{i}\log(\beta_{\mathrm{loc}}x_{i,\mathrm{loc}}(t)+\beta_{\mathrm{mob}}x_{i,\mathrm{mob}}(t))-\log((-\Delta S_{i}(t))!)\\ & \;\;+\log(\Gamma (r-\Delta S_{i}(t)))-\log(\Gamma (r))+\Delta S_{i}(t)\\ & \;\;\;\log(r+\beta_{\mathrm{loc}}x_{i,\mathrm{loc}}(t)+\beta_{\mathrm{mob}}x_{i,\mathrm{mob}}(t))\\ & \;\;-r\log\left(1+\frac{\beta_{\mathrm{loc}}x_{i,\mathrm{loc}}(t)+\beta_{\mathrm{mob}}x_{i,\mathrm{mob}}(t)}{r}\right)), \end{array}$$

where Γ is the gamma function and where the *i*th elements of the vectors *x*_loc_(*t*) and *x*_mob_(*t*) are given by

$$x_{i,\mathrm{loc}}(t)\;:=\frac{S_{i}(t)}{N_{i}}(I_{i}^{T}(t)+I_{i}^{U}(t))$$

and

$$x_{i,\mathrm{mob}}(t)\;:=\sum_{\;j\in D}\frac{S_{i}(t)}{N_{i}}\frac{I_{j}^{U}(t)}{N_{j}}(M_{\;ji}+M_{ij}).$$

The desired estimate $\left({\hat{\beta }}_{\mathrm{loc}},{\hat{\beta }}_{\mathrm{mob}}\right)$ of (*β*_loc_, *β*_mob_) and an estimate $\hat{r}$ of the corresponding *r* is a maximizer of this function wherein we substitute our estimates for the compartments and mobility data as described in §3. Since *L*_NB_ is concave in *β*_loc_, *β*_mob_ and *r*, standard descent algorithms can be employed to find such a maximizer efficiently. The confidence intervals for the estimates are computed via parametric bootstrapping (see electronic supplementary material, appendix E).

One consequence of estimating the transmission rates in this way is that the terms ${\hat{\beta }}_{\mathrm{mob}}x_{i,\mathrm{mob}}$ are invariant to the scale of *M*. Thus, when the used proxy $\hat{M}$ for *M* has the same structure as *M* but differs primarily in the volume, the estimated transmission rates can be scaled to obtain suitable estimates for the transmission rates obtained when having used the true mobility matrix *M*. This also holds for the resulting estimate of the fraction of local contacts *p*. Note that this observation is independent of the data source used for the proxy $\hat{M}$. This observation explains why our methods are not very sensitive to changes in mobility volumes.

### 4.2. Validation on the national level

The model validation consists of two steps. In each step, we compare an output of the model to the corresponding reported values by RIVM [30]. The two considered outputs are the daily reported positive tests and the effective reproduction number. Within our model, the number of daily reported positive tests for a given day *t* is represented via equation (2.2) by the term $\sum_{i\in D}a({\hat{E}}_{i}(t)/\nu )$. We focus on two methods for calculating this term from our model:

(1) The first method is based purely on the initialization approach. More precisely, we directly compute for each day *t* the corresponding term as
  $$\sum_{i\in D}a\frac{{\hat{E}}_{i}(t)}{\nu }=\frac{1}{\nu }\int_{0}^{\infty }\;e^{-(s/\nu )}\Delta I_{i}(t+s)\;ds$$
  (see also electronic supplementary material, appendix B). This method allows us to check the validity of the initialization approach.
(2) In the second method, for each day we first estimate the transmission rates as described in §4.1. Subsequently, we initialize the compartments at the first date, 1 July, and simulate the model until the last day, 31 December, using for each day the corresponding estimated transmission rates, fraction of positive tests, and mobility data as input. This procedure directly yields the desired terms for each simulated date. To reduce the influence of propagating errors, we re-initialize the compartments at every first day of the month. This method allows us to check the validity of the model dynamics, i.e. whether the model is able to adequately capture the development of new positive cases over time.

The effective reproduction number is computed in our model by constructing the *next-generation matrix* (NGM) [40] based on our model and estimates of the transmission rates (for more details on the construction of this matrix, we refer to [3]). If the general trend of our and RIVM’s estimate matches, then the underlying methods to obtain these estimates agree on the representation of the dynamics between susceptible and infectious individuals (see [41,42] for a description of the estimation methods used by RIVM).

However, we also expect that our estimates may have a delay of several days for two reasons. First, in our model we do not account for the time between planning a test appointment (for example, because one develops symptoms) and receiving a (positive) test result. However, throughout the studied time period 1 July–31 December, the average of this time has varied between 35 and 81 h in The Netherlands [43]. As a consequence, we might estimate the number of infectious and tested individuals with some delay. By contrast, the calculation method used by RIVM does allow for delays in testing and reporting [41,42].

The second reason is that our computation of the effective reproduction number via the NGM and estimated transmission rates uses information on daily reported positive cases from both before and after the date as input. On the other hand, the aforementioned calculation method of RIVM uses detailed information about new reported hospital admissions. These are reported by hospitals with some delay and these numbers represent infections that took place up to 14 days previously. One reason that the RIVM uses hospital admissions is that the daily numbers of positive tests depend on the number of people that get tested and this number varies also for reasons other than variation in new cases (for example, changes in testing strategy in the early phases of the outbreak in The Netherlands).

To assess whether our estimates have some delay compared to the estimates of RIVM, we determine the amount of days by which we should shift our estimates as to obtain the largest correlation between these time-shifted estimates and those of RIVM.

### 4.3. Forecast quality

We compute forecasts of the number of newly reported infections in the next 14 days, i.e. the 14-day incidence. To obtain these forecasts, we initialize the compartmental model 7 days in the past and simulate it over a horizon of 21 days (recall that we cannot initialize the model for the current date since the number of exposed people is calculated from the number of reported cases within the next 7 days). We choose the necessary parameters *β*_loc_, *β*_mob_ and *a* as the average of these parameters over the past 7 days from the start of the forecast horizon. As an example, suppose that the current date is 11 August. Then we aim to make forecasts of the number of newly reported infections within the period 11–25 August and initialize the model on 4 August using as parameter inputs the averages of these parameters over the period 28 July–3 August.

We distinguish between the absolute and relative number of reported positive cases, where the relative number is in comparison to the total nationwide number of infections. The latter values provide insight into the risk that infections will occur in a given municipality. Thus, they are useful risk information for local policymakers.

With respect to the absolute number of positive tests, we directly compare our forecasts of the 14-day incidence to the actual 14-day incidence within the chosen period, both on the national level and that of municipalities. With regard to the level of municipalities, we assess whether there is a spatial discrepancy in forecast accuracy between different municipalities.

With respect to the relative number of positive tests, we investigate whether we can accurately forecast the *order* of municipalities in terms of the 14-day incidence. We do this by calculating the correlation between the *orders* of the forecast and observed fractions of 14-day incidence via the Spearman correlation.

### 4.4. Influence of mobility

We assess the influence of mobility in two ways. First, we investigate under which conditions including mobility leads to a better fit of the transmission rates. To this end, we consider two different models for estimating the transmission rates via MLE, which are given by the presence or absence of mobility. When estimating the rates when mobility is excluded, we set *β*_mob_ = 0 in the expressions for the log-likelihood function in equation (4.2). To assess which of these models fits the data best, we compute and compare for both models the Akaike information criterion (AIC) [44]. For each model, this criterion is computed as $2k-2\log\hat{L}$, where *k* is the number of unknown parameters that the model estimates and $\log\hat{L}$ is the maximum value of the log-likelihood function. A lower AIC implies that the given model fits the data better. In particular, a difference in the AIC of two models of more than 10 is generally considered to be a strong indication that the model with the lower AIC value is a better fit to the data [45].

Second, we investigate under which conditions the inclusion of mobility leads to more accurate forecasts of the number of reported positive tests. For this, we compute for both model versions (including or excluding mobility) forecasts as described in the previous subsection and compare the difference in accuracy by means of the mean absolute error (MAE) (for the absolute number of infections) and the Spearman correlation (for the relative number of infections).

## 5. Results

We now present and discuss the results of the estimations and experiments described in the previous section.^1^ To assess the influence of new measures on the estimated parameters and on the forecasting accuracy, we provide an overview of the restrictive measures taken in The Netherlands within the studied time period in table 2. In all figures in this section, the dates at which these measures were installed are indicated by green (relaxation), orange (restriction) and red (lockdown) vertical lines.

**Table 2.** Overview of measures taken in The Netherlands between 1 July and 31 December 2020. Adapted from [46].

date	type	description
1 July	relaxation	only basic restrictions (keeping 1.5 m distance, washing hands, etc.)
6 August	restriction	stricter rules for attending public places (e.g. mandatory pre-registration)
18 August	restriction	at most six guests at home
29 September	restriction	at most three guests at home, bars and restaurants close at 22.00 h, no audience at sport events; advice to work from home
14 October	partial lockdown	bars and restaurants closed, no events, face masks mandatory at schools, sport in groups of at most four; strong advice to work from home
4 November	partial lockdown	at most two guests at home, public places closed, sport in groups of at most two
14 December	lockdown	schools, non-essential stores and gyms closed
24–26 December	temporary relaxation	at most three guests at home

### 5.1. Estimation of the transmission rates and related parameters

**Figure 1.**
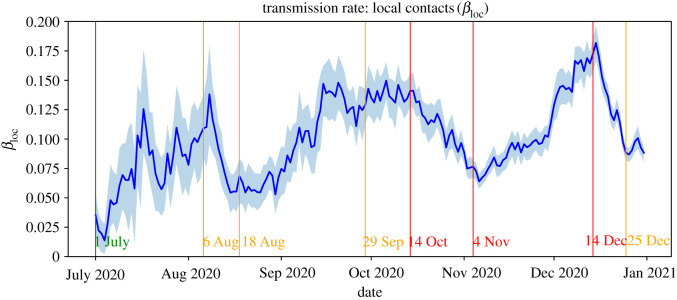
Estimations of the local transmission rate *β*_loc_ with 95% confidence interval.

**Figure 2.**
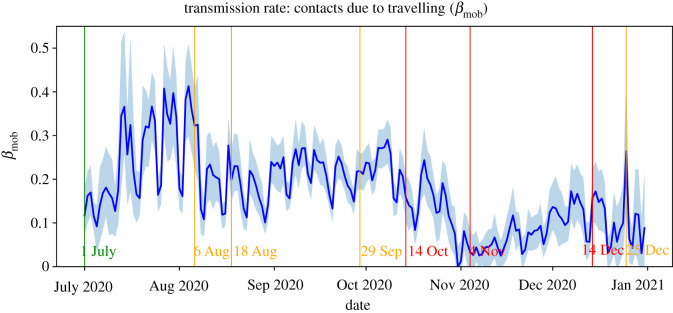
Estimations of the mobility-induced transmission rate *β*_mob_ with 95% confidence interval.

Figures 1 and 2 show the estimates of the transmission rates *β*_loc_ and *β*_mob_. The downward peaks in *β*_mob_ correspond to weekend days where the volume of commuting traffic is significantly lower than on weekdays. Generally, the behaviour of the transmission rates matches in the sense that they increase and decrease in parallel. A notable exception to this can be observed in the period 18 August–29 September, where *β*_loc_ increases steeply and *β*_mob_ remains relatively stable. This behaviour is in line with schools starting again in September after the summer break, which led to an increase in local contacts among school children and their teachers and parents.

**Figure 3.**
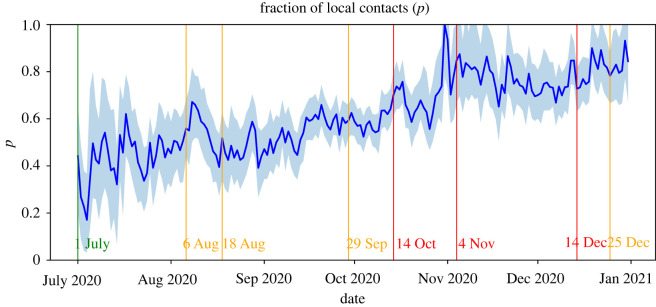
Estimations of the fraction *p* of local contacts with 95% confidence interval.

Figure 3 shows the estimated values for the fraction *p* of local contacts. This fraction appears to increase steadily throughout the considered time period, which is in line with the increasing degree of mobility-restricting measures. In particular, a sudden increase occurs shortly after the partial lockdown restrictions of 4 November. This suggests that our estimates capture the logical trend with respect to these measures.

**Figure 4.**
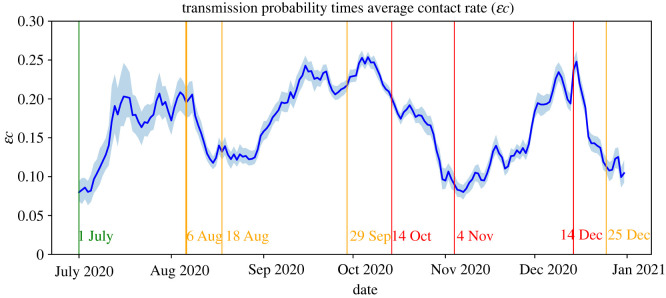
Estimate of the term ɛ*c* (transmission probability times average contact rate) with 95% confidence interval.

Figure 4 shows the estimate of the term ɛ*c*, i.e. the transmission probability times the average contact rate. Although ɛ and *c* cannot be estimated separately when none of them is given on forehand (see equations (2.2) and (2.3)), the estimated term ɛ*c* does provide information on changes in the number of contacts, assuming that the transmission probability remains constant over time. For instance, it follows from figure 4 that the number of contacts has increased following the relaxation of measures after 1 July, following the start of the new school year in September, and following the partial lockdown measures after 4 November. Moreover, after the restrictions on 6 August and the (partial) lockdown measures of 14 October and 14 December, the number of contacts has decreased. This is in line with the governmental-issued advice to work from home in this period, which led to a reduction in commuting travel.

### 5.2. Validation results

**Figure 5.**
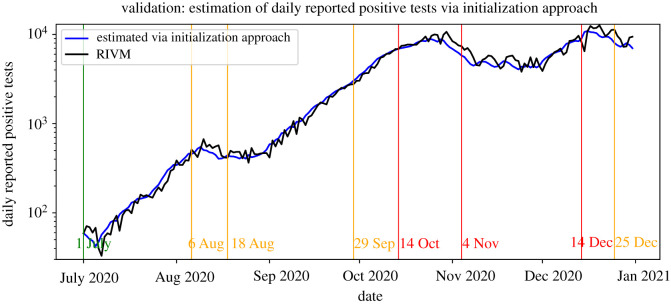
Validation of the initialization method.

**Figure 6.**
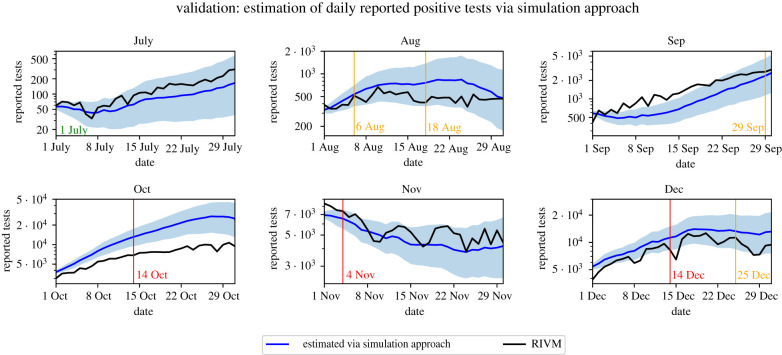
Validation of the simulation method with 95% confidence intervals.

Figures 5 and 6 show the estimates of the daily reported positive tests computed via the initialization and simulation method, respectively (see §4.2), and those as reported by RIVM. Figure 5 indicates that the estimation via the initialization method closely follows the positive tests as reported by RIVM. This suggests that our initialization approach is able to estimate the state of the epidemic well on the national level. Figure 6 indicates that the simulation method is generally able to follow the general trend of the reported new tests. This suggests that our model and our methods for parameter initialization and estimation are successful in capturing the development of the number of daily reported positive tests on the national level. Moreover, our results suggest that the used data sources on commuting mobility and SARS-CoV-2 seroprevalence can be integrated successfully into compartmental models.

To determine whether our estimates of the effective reproduction number have a time delay compared to those of RIVM, we computed the correlation between these two estimates for different numbers of delay of days. These results show that there is a strong correlation between these estimates when the delay is between 5–14 days and is the strongest for a delay of 11 days (0.865). This is in line with the reporting delay and difference in time window and data sources between our method and the methods used by RIVM.

**Figure 7.**
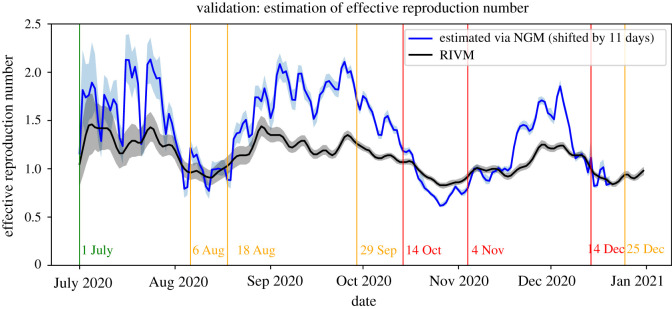
The effective reproduction number as estimated in this paper (shifted by 11 days) and by RIVM with 95% confidence intervals.

Figure 7 shows the estimates for the effective reproduction number, shifted by 11 days, and the estimates made by RIVM. Overall, our estimates appear to be somewhat more extreme than those of RIVM. However, both estimates share the same general pattern with regard to periods of monotonic increases and decreases and agree on whether the epidemic is expanding or dying out, i.e. whether the effective reproduction number is larger or smaller than 1. Moreover, the peaks and valleys in the estimates match very well, which confirms the relative consistency of the estimates and the presence of a temporal shift. Overall, the validation shows that our estimation method agrees qualitatively with the method used by RIVM.

### 5.3. Forecast accuracy

**Figure 8.**
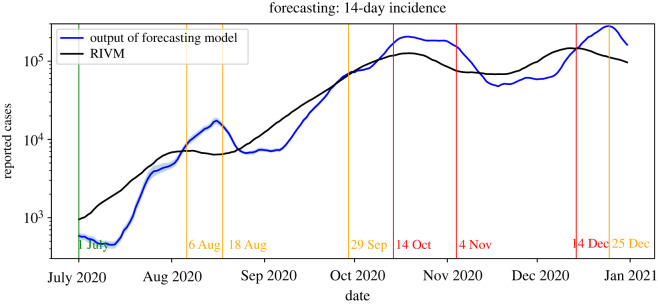
Forecast and actual incidence within the following 14 days with 95% confidence interval.

We first focus on the accuracy of our forecasts of the *absolute* number of daily reported cases. Figure 8 shows for each date within the period 1 July–31 December 2020, our forecast and the actual number of newly reported cases nationwide within the following 14 days. This figure shows that, generally, the forecasts follow the actual 14-day incidence. Analogous to the estimation of the effective reproduction number, it appears that the forecasts have a certain delay. One reason for this is that our model is necessarily initialized using parameters from one to two weeks ago as input. When the transmission rates are relatively stable for a longer period of time, this effect is minimal. In this case, the forecast error is relatively small (e.g. around 1 August and in October). However, when the transmission rates are not stable, for example due to newly introduced preventive measures, the forecasts are made using data that are not representative of the current epidemiological situation. Consequently, it takes some time before the input data of the forecast procedure is again a proper representation of the future parameter values (see also the behaviour of the transmission rates in figures 1 and 2).

**Figure 9.**
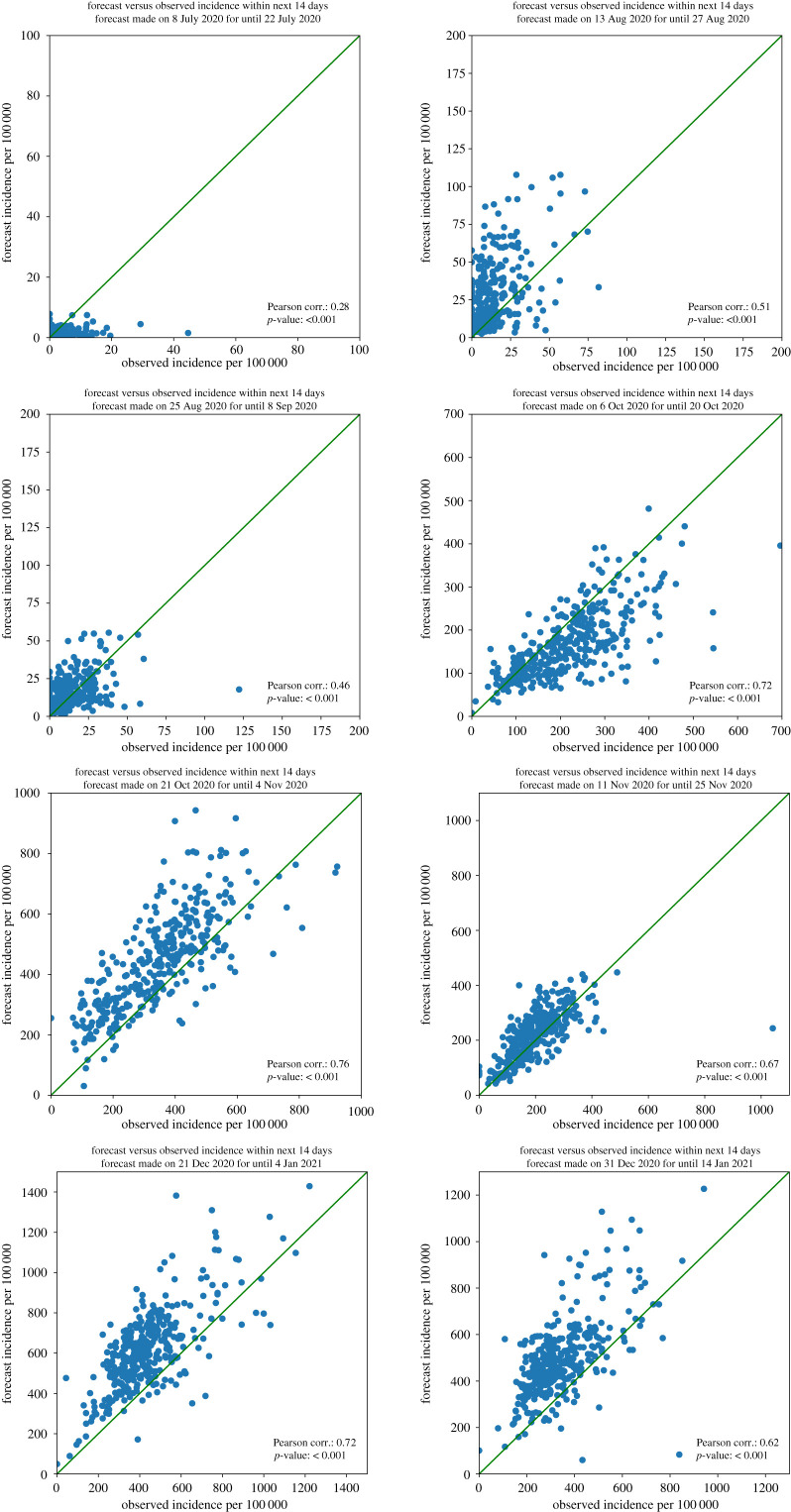
Scatter-plots of the accuracy of forecasting 14 days ahead (taking mobility into account) starting from the eight selected dates.

**Figure 10.**
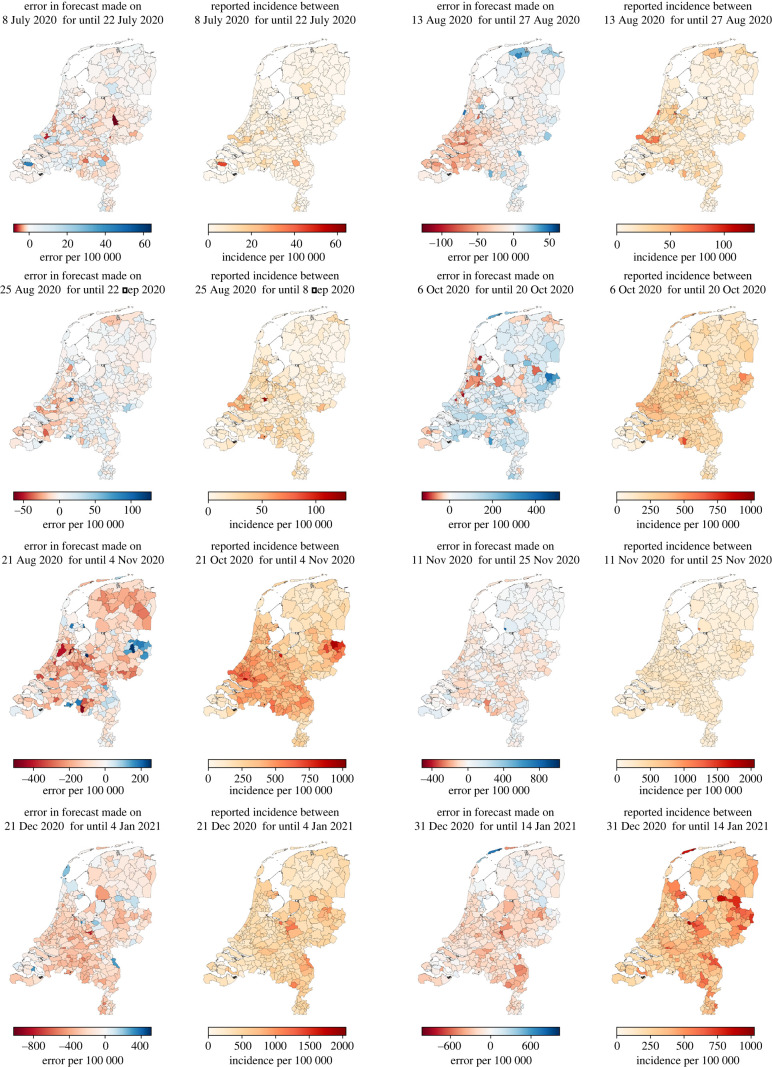
Geographical representation of the error in forecasting 14 days ahead (taking mobility into account) starting from the eight selected dates.

We now focus on the forecast accuracy at the level of municipalities. First, we consider differences in accuracy over *time*, by discussing the results for eight specific dates, namely those that are one week after a change in preventive measures as indicated in table 2: 8 July, 13 August, 25 August, 6 October, 21 October, 11 November, 21 December and 31 December (see the electronic supplementary material for the results of all the other dates). For each of these eight dates, figure 9 shows for all 355 Dutch municipalities, each of which represented by one dot, the forecast and actual 14-day incidence within that municipality over the next 14 days combined. Moreover, figure 10 shows the forecast error and, as a scale reference, the actual 14-day incidence starting from these dates.

These figures confirm the observation made earlier on the national level that our forecasts are relatively accurate for periods where the transmission rates are stable. For instance, this is the case for the forecast for 11–25 November but not for the forecast for 21 October–4 November. Furthermore, the maps suggest that, in most cases, there are only a few municipalities with a relatively large forecast error. Thus, generally, the forecast error is distributed quite evenly over the municipalities. A notable exception to this is the forecast for 21 October–4 November, which can be explained by the relative instability of the corresponding transmission rates.

The graphs in figure 9 suggest that the variation in forecast error is quite high for 8 July, 13 August and 25 August, and relatively small for the other five dates. One explanation for this is that the actual numbers of daily new infections was relatively small compared with the other dates. In particular, many municipalities had only a few or even zero reported cases within the considered time periods, meaning that adjusting a small forecast error for population size may result in large differences per 100 000 inhabitants.

**Figure 11.**
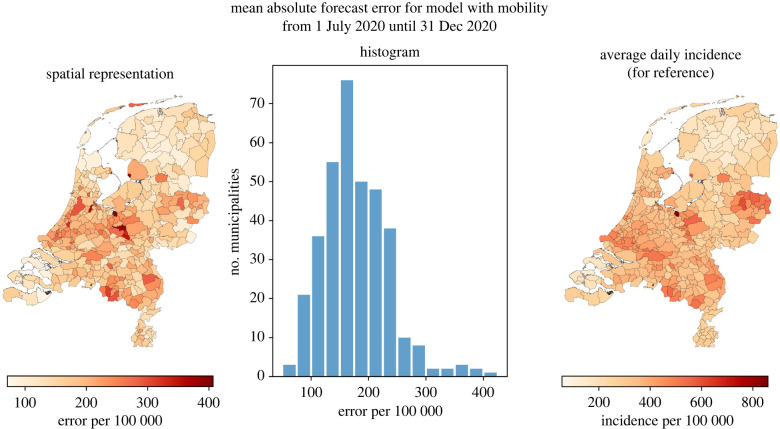
Mean absolute error of the 14-days ahead forecast, evaluated per municipality.

To identify potential structural differences in forecast accuracy between municipalities, figure 11 shows the average absolute error over the entire time horizon for each municipality (left map and histogram) and, as a scale reference, the average daily incidence (right map). We found a moderate correlation between the absolute error and total number of reported positive cases (Pearson’s *r* = 0.71, *p* < 0.001, *n* = 355). This suggests that, overall, the error is larger for municipalities that have had disproportionately many positive cases, which is also visible from the maps in figure 11. However, as visible from figure 10, this does not mean that there is always a strong relation between large numbers of infections and a large forecast error on a day-to-day basis. For instance, for 21 October, large forecast errors occur for ranges of municipalities with both low incidence numbers (e.g. in the northern municipalities) or high incidence numbers (e.g. in the central eastern municipalities).

Municipalities with a relatively high error are in some sense distinct in terms of the number of contacts that they have and are different from the average municipality. Based on figure 11, we highlight two of such distinct groups of municipalities.

— First, we observe that the error is relatively high among municipalities in the so-called ‘Bible-belt’. This is a non-administrative area stretching from the southwest towards the northeast and has the highest concentration of conservative orthodox Protestants in the country [47]. In particular, the five municipalities with the highest average error are all part of this area. This observation is in line with other research on the positive relation between the level of church involvement and virus transmission within communities [48].
— Second, the prediction errors in municipalities at international borders (in the south and east) seem generally higher than those of their neighbouring municipalities that do not lie at such a border. This suggests a significant influence of foreign mobility on transmissions in these municipalities. Interestingly, this also applies to the municipality Haarlemmermeer that houses the main international airport in The Netherlands (Amsterdam Airport Schiphol) and ranks 12th in terms of highest average error.

**Figure 12.**
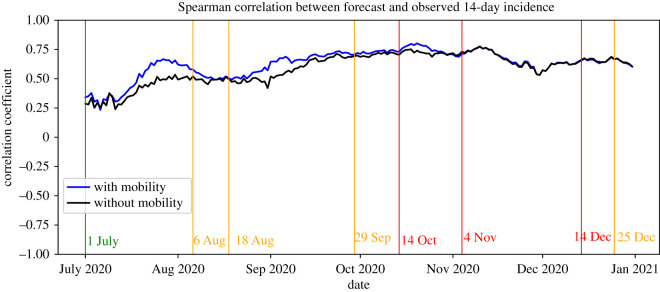
Correlation between the orders of the 14-days ahead forecast and observed fractions of reported infections.

We now assess the quality of our relative forecasts, i.e. forecasts of the fraction of reported infections in a given municipality compared to the nationwide number. The blue line in figure 12 shows the Spearman correlation between the forecast and actually reported 14-day incidence. Note that a correlation of 1 or −1 means that the order of the fractions as forecast by our model is the same as, or the reverse of, that of the observed fractions, respectively. The calculated values in figure 12 indicate that, apart from a small period at the start of July, the two orders are moderately and sometimes even strongly correlated (from mid-September until mid-November). This suggests that our approach is able to forecast the order of municipalities in terms of new infections with reasonable accuracy.

### 5.4. Influence of mobility

**Figure 13.**
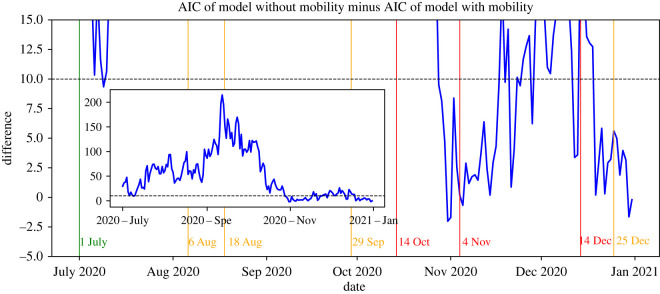
The difference in AIC for the models with and without mobility (zoomed in for differences between −5 and 15).

Figure 13 shows the AIC of the negative binomial model without mobility minus that of the model with mobility. For clarity, we have zoomed into a small band around the critical zero point that determines which of the models has a better fit. These results show that the differences in AIC are generally larger than 10 before the partial lockdown initiated on 14 October. This suggests that, until that day, incorporating mobility in the estimation process leads to a significantly better fit. However, throughout the remainder of the year, the differences are generally between −2.5 and 10, meaning that for this period the fit may not significantly improve when mobility is taken into account. Note that this period matches almost perfectly with the time when there was an urgent advice to work from home. Therefore, these observations suggest that taking into account information on commuting mobility does not lead to a significantly better model fit when there is such a strong advice in place.

**Figure 14.**
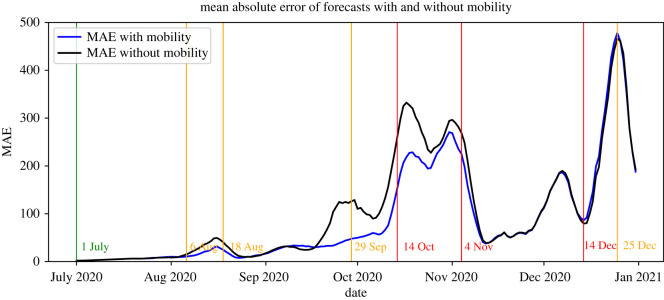
MAE of the 14-days ahead forecasts (not per 100 000) of the models with and without mobility.

To assess the additional value of taking mobility into account when forecasting the absolute numbers of new infections, we calculate for each date the MAE of the corresponding forecast for both models and compare these values in figure 14. These results indicate that in most cases the forecasts that incorporate mobility are more accurate than those that do not. Moreover, after the partial lockdown measures of 4 November, the MAE of both models is practically equal. This suggests that the influence of mobility on forecast accuracy is insignificant in this period.

**Figure 15.**
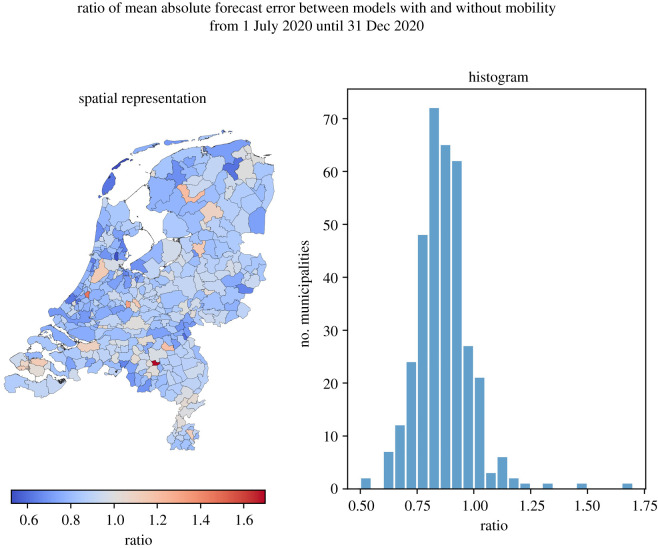
Ratio between the MAE of the 14-days ahead forecasts of the models with and without mobility, per municipality.

To investigate the improvement in forecast accuracy on the municipality level, for each municipality we calculate the mean absolute error of the corresponding forecast over the entire time horizon for both models and compare these values in figure 15. These results suggest that for almost 90% of the municipalities (319 out of 355), including mobility leads to an overall improvement in forecast quality. The remaining 36 municipalities do not appear to display a clear connection or pattern in this regard. Furthermore, we did not find any significant correlation between this ratio and absolute mobility volumes. We do, however, find that the ratio correlates moderately with the average daily incoming mobility *relative to the number of inhabitants* (*r*(353) = 0.56, *p* < 0.001). This suggests that the inclusion of mobility may improve the forecasts particularly for municipalities where we would expect relatively much incoming commuting travel.

With regard to the forecast accuracy in the relative number of new infections, we again consider the Spearman correlations in figure 12. In essentially all cases, the correlation for the model with mobility is higher than that for the model without mobility. This means that taking mobility into account generally leads to better forecasts of the relative order of municipalities in terms of new infections. This suggests that taking mobility into account also improves the ability of our approach to identify local outbreaks. Furthermore, the correlations are practically the same in the period November–December, meaning that taking mobility into account does not significantly improve the forecasts in this period. This is consistent with the behaviour of the MAE as discussed above.

## 6. Conclusion and discussion

In this paper, we studied the transmission of SARS-CoV-2 in The Netherlands during the first year of the pandemic and the role of mobility on this transmission. To model transmissions between different regions, we employed the mobility-enhanced SEIR-compartmental model in [3]. We obtained suitable parameter values for this model using commuter data and information on the seroprevalence of SARS-CoV-2. In particular, we developed an MLE approach to fit suitable transmission rates for the compartmental model. Using the initialized model, we are able to provide accurate forecasts of the development of the transmission on the level of municipalities for up to 14 days.

We found that taking mobility into account generally improves the model fit and forecast quality, but that this improvement is insignificant when mobility and contacts are restricted by national preventive measures. More precisely, we observed that the partial lockdown measures of 4 November 2020 led to sudden changes in parameter estimates and forecast accuracy. These observations suggest that these measures were particularly effective in reducing contacts and more so than those of the first partial lockdown on 14 October 2020. In particular, the closing of public places might have forced a significant part of the working population to work from home again. Such a change in mobility would be detected directly by the current model since the used mobility data consist purely of commuting information.

Many of the municipalities with an exceptionally high error are part of the so-called Dutch ‘Bible belt’ which may have a local contact structure and intensity that deviates substantially from the average. Moreover, municipalities with a higher level of international travel, for instance at the border, seem to have a higher forecast error on average than their direct neighbours that are not at the border. These observations suggest that there is a certain heterogeneity in terms of the number of contacts that is not captured by the current model. An initial intuitive approach to account for this heterogeneity could be to introduce separate contact rates for each municipality. However, as we show in electronic supplementary material, appendix F, this cannot be achieved by simply extending the currently used mobility-enhanced compartmental model.

We observe that the estimated parameters and forecast accuracy of the model generally remain relatively stable during periods where no new measures are being enforced. However, our work also shows that this changes dramatically when there is a change in mobility-restricting measures since the historic data used for initialization are no longer representative for the future. One way to correct for this, which we also aim to address in future work, is to integrate the most recent information on people’s behaviour into the estimation procedure, which could be obtained from, e.g. contact tracing apps.

We conclude by discussing several limitations of the research in this paper. First, we focus solely on inter-regional mobility and do not take other traits into account that influence susceptibility, infectivity, contact patterns and virus transmission, such as age, behaviour or household composition, into account. In comparison, the models used by the national public health institute RIVM take the latter traits into account, but do not explicitly include mobility. Thus, although our focus on inter-regional mobility is a limit, it does complement the focus of the models by RIVM. Also, we focus on the early phases of the pandemic, when vaccines were not yet available and hence the proportion of people who were protected was relatively small and only resulting from natural infection. It would be easy to add vaccination to the model, but it would add yet another layer of complexity because one would need to include how vaccinated individuals change their susceptibility, infectivity, contacts and mobility. Additionally, as vaccinations increased, adherence to preventive measures changed in the population, as well as testing strategy and willingness to get tested.

Second, we use only commuter travel information as a mobility proxy. This is a limitation because we cannot test hypotheses and draw conclusions on general relations between mobility and, say, forecast quality. We also do not take other reasons for travel and travel destinations other than workplaces into account where transmission can occur such as social gatherings, mass events and holidays. Moreover, with regard to the used mobility data, the combination of pre-pandemic commuting information and relative changes in workplace mobility yields a limitation since the latter is not given per pair of origin-destination municipalities but only per municipality.

To obtain a more realistic proxy for the mobility data, sources other than commuter information should be incorporated as well. One option for this is travel behaviour obtained via track-and-trace apps or location data obtained from cellphones [49]. However, these data typically contain privacy-sensitive information, for which the risk exists that observed movements are traced back to individual users. Therefore, these data must be sufficiently anonymized and/or aggregated to circumvent this issue. However, even when the data are anonymized, often permission for its usage is required. Summarizing, it is difficult, if not impossible, to obtain such detailed data. Therefore, one reasonable question for future research is how alternative data sources may be used to obtain suitable proxies for the actual mobility.

Finally, a similar problem occurs when certain epidemiological data are not available or no longer representative. For instance, an increase in vaccinated individuals leads to a decrease in hospitalizations. As a consequence, the difference between the number of new infections and new hospitalizations becomes larger, meaning that the latter becomes less representative of the number of infectious people. Since the latter is used to initialize a crucial parameter in our model (namely the fraction of positively tested infectious people), we expect the performance of the model and our initialization procedure in its current form to decrease when applied to a time period when a substantial part of the population has been vaccinated. One way to solve this issue could be to estimate the number of infectious people using other statistics that are known to correlate with this information, such as the daily fraction of positive tests.

## Data Availability

All used data and source code are freely accessible. The data on reported COVID-19 cases were obtained from the Dutch National Institute for Public Health and the Environment (RIVM) via https://data.rivm.nl/covid-19/. The commuting data were obtained from Statistics Netherlands (CBS) via https://opendata.cbs.nl/#/CBS/nl/dataset/83628NED/table?dl=489D. The data on relative changes in mobility were obtained from Google LLC via www.google.com/covid19/mobility/. The source code that was used to conduct the numerical experiments is available at https://github.com/MartijnGosgens/mobility-forecasting-covid. Electronic supplementary material is available online [50].

## Footnotes

1 The corresponding source code is available at https://github.com/MartijnGosgens/mobility-forecasting-covid.
